# The Role of Ubiquitination in Regulating Embryonic Stem Cell Maintenance and Cancer Development

**DOI:** 10.3390/ijms20112667

**Published:** 2019-05-30

**Authors:** Dian Wang, Fan Bu, Weiwei Zhang

**Affiliations:** College of Life Sciences, The Capital Normal University, 105 Xi San Huan Bei Road, Hai Dian District, #322, Science Building, Beijing 100048, China; 2180802096@cnu.edu.cn (D.W.); bufan0325@126.com (F.B.)

**Keywords:** ubiquitination, embryonic stem cells, colorectal cancer, osteosarcoma

## Abstract

Ubiquitination regulates nearly every aspect of cellular events in eukaryotes. It modifies intracellular proteins with 76-amino acid polypeptide ubiquitin (Ub) and destines them for proteolysis or activity alteration. Ubiquitination is generally achieved by a tri-enzyme machinery involving ubiquitin activating enzymes (E1), ubiquitin conjugating enzymes (E2) and ubiquitin ligases (E3). E1 activates Ub and transfers it to the active cysteine site of E2 via a transesterification reaction. E3 coordinates with E2 to mediate isopeptide bond formation between Ub and substrate protein. The E1-E2-E3 cascade can create diverse types of Ub modifications, hence effecting distinct outcomes on the substrate proteins. Dysregulation of ubiquitination results in severe consequences and human diseases. There include cancers, developmental defects and immune disorders. In this review, we provide an overview of the ubiquitination machinery and discuss the recent progresses in the ubiquitination-mediated regulation of embryonic stem cell maintenance and cancer biology.

## 1. Introduction

Ubiquitination is an energy-dependent enzymatic process to modify proteins with a small-sized polypeptide ubiquitin (Ub) at the post-translational level. Its name was derived from the Latin “ubique”, which means "everywhere", describing its ubiquitous expression pattern in diverse types of eukaryotic cells [1]. Ub is highly conserved in sequence and structure across animals, plants and fungi [2,3]. It modifies thousands of intracellular proteins either as a "death label" for degradation or non-proteolytic signal governing protein activity [4,5,6,7]. This modification is of great biological significance since it encompasses nearly all aspects of cellular events. Dysregulation in ubiquitination leads to severe consequences and human diseases, such as cancers, degenerative diseases and immune disorders [8]. This review provides overview of the enzymatic machinery mediating ubiquitination and surveys the roles of ubiquitination in regulating embryonic stem (ES) cell maintenance and cancer development.

## 2. The Ubiquitination Machinery 

Canonical ubiquitination is an ATP-dependent enzymatic process during which an isopeptide bond is formed between the C-terminal carboxy group of the Ub residue glycine-76 (Ub-G76) and the ε-amino group of lysine (K) residues in proteins. In a small number of instances, Ub can be conjugated to the N-terminal methionine (M1) and other nonlysine residues, such as cysteine (C), serine (S), threonine (T) or tyrosine (Y) [9,10,11,12,13,14]. For instance, members of the SidE family, which serve as effectors of the pathogen *Legionella pneumophila*, can directly ubiquitinate the S residues of substrates via the phosphor-ribosyl linkage [15]. 

**Figure 1:**
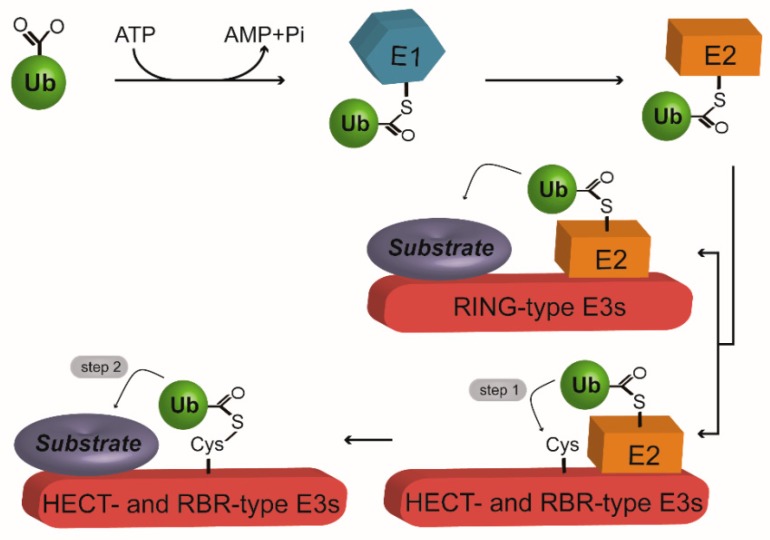
The ubiquitination machinery. Ubiquitination is initiated by E1-mediated ubiquitin (Ub) activation. Next, Ub is transferred to E2 to form an E2-Ub conjugate. At the final step, E3 mediates isopeptide bond formation between the Ub and the substrate. Really interesting new gene (RING)-type E3s serve as a scaffold to directly transfer the Ub from E2 to the substrate. On the other hand, homologous to E6-AP COOH terminus (HECT)- and RING between RING (RBR)-type E3s require a two-step reaction to achieve Ub ligation with the substrate. In the first step, Ub is transferred from E2 to E3, producing an E3-Ub thioester intermediate. At the second step, Ub is finally handed over to the substrate. Arrows represent the next steps during the process of ubiquitination.

Ubiquitination is generally achieved by the machinery comprising three types of enzymes (Figure 1). They include Ub-activating enzyme (Uba, E1), Ub-conjugating enzyme (UBC, E2) and Ub ligase (E3) [4]. The catalytic activities of these enzymes are sequentially organized in the reaction cascade. Firstly, E1 activates the Ub-G76 residue in ATP hydrolysis-dependent manner to produce a Ub-adenylate intermediate. Subsequently, AMP is released and the Ub is transferred to the active C site of E1 via a thioester bond [16]. Next, the E1-Ub conjugate interacts with E2 for a transthiolation reaction during which the E1- activated Ub is transferred to the active C site of E2 to form an E2-Ub conjugate. At the final step, E3 concurrently associates with the E2-Ub conjugate and a substrate protein to mediate isopeptide bond formation between the Ub moiety and substrate [17]. In general, E2s dominantly determine the exact K residue of Ub for ubiquitination, the number of Ub moieties added and the linkage type of poly-Ub chains, whereas E3s regulate the specificity of substrate recognition [18,19,20,21]. Although the E1-E2-E3 cascade is adopted by most intracellular processes, some instances require an additional E4 ligase for poly-Ub chain extension or multi-Ub chain assembly [22,23,24,25,26,27,28,29]. For example, E3 mouse double minute 2 homolog (MDM2) modifies p53 with a single Ub moiety (monoubiquitination). Following that, p300 serves as an E4 ligase, adding more Ub moieties to the monoubiquitinated residue of p53 to form a poly-Ub chain [29]. Of note, ubiquitination can also be accomplished by a single E3, in place of a multi-enzyme machinery. Such fact has been reported for the mono-ADP-ribosyltransferase SdeA. SdeA is secreted by *Legionella pneumophila* in the host cells. To directly ubiquitinate host's substrate proteins, it utilizes nicotinamide adenine dinucleotide (NADH) to form ADP-ribosylated Ub [30]. 

Compared with E1s, there is a wider variety of E2 and E3 enzymes in eukaryotes. The human genome encodes only two E1s, but 40 E2s and over 600 E3s [20,21,31]. All E2s contain a conserved catalytic UBC domain with the active site C. The UBC domain has about 150 amino acids and constitutes the full-length sequence of class I E2s. In addition, other E2s possess extended sequences at either the C- (class II) or the N-terminus (class III). Meanwhile, E2s with extension regions at both the N- and C-terminus are grouped as class IV. The extension regions are involved in the determination of cellular localization and protein-protein interaction [31,32].

E3s are the most abundant enzymes involved in ubiquitination. According to their catalytic domains and Ub transfer mechanisms, E3s are classified into three groups. These comprise of the Really Interesting New Gene (RING)-type, homologous to E6-AP COOH terminus (HECT)-type and RING between RING (RBR)-type E3s [33]. The RING-type E3 family members are characterized by its RING or U-box domain. These two domains exhibit similar RING finger fold in structure. However, the activity of RING domain requires chelation of two zinc ions (Zn2+), whereas the U-box domain is Zn2+-independent. During ubiquitination, RING-type E3s serve as a scaffold for the binding of the E2s and their substrates. This allosterically stimulates a direct transfer of Ub moiety from the E2-Ub conjugate to the substrates [33]. Compared with the other types of E3s, RING-type E3s represent the most abundant ligases with over 500 family members [33]. Notably, some RING-type E3s, also known as the Cullin-RING ligases (CRLs), form a large complex with multiple subunits to mediate ubiquitination [34]. In spite of its diversity in subunit assembly, all CRLs possess at least four common subunits, including an E2-binding catalytic RING finger, a scaffold comprising seven Cullins (CUL1, CUL2, CUL3, CUL4A, CUL4B, CUL5, and CUL7), a receptor for substrate recognition and an adaptor arm responsible for the linkage between the receptor and the Cullin scaffold [34]. Two typical CRLs are the anaphase-promoting complex/cyclosome (APC/C) and the Skp1/Cul1/F-box (SCF). 1.2 MDa-sized APC/C is a large ligase complex which consists of 19 subunits, such as the Apc11 (RING subunit), Apc2 (Cullin scaffold) and coactivator subunit Cdc20/Cdh1 [35,36]. Apc11 and Apc2 form the catalytic center, while Cdc20/Cdh1 is involved in substrate recognition and enhancement of the catalytic activity of Apc11 [35,37]. The HECT-type E3s possess a conserved catalytic HECT domain with the active site C at the C-terminus and a variable N-terminal extension that largely determines the specificity of its substrate recognition [34]. There are about 28 HECT-type E3s encoded by the human genome [38]. According to the variable N-terminal extensions, these HECT-type E3s can be further classified into three subfamilies, including the WW domain-containing Nedd4/Nedd4-like E3s, HECT and RCC1-like (HERC)- and RCC1-like domains (RLD)-containing E3s, and the HECT-type E3s without WW and RLD domains [39]. Distinct from the RING-type ligases, HECT E3s require a two-step reaction to ligate Ub with sustrates. In the first step, the Ub moiety from the Ub-E2 conjugate is transferred to the catalytic C site of HECT-type E3 to form a HECT-Ub thioester intermediate. Subsequently, the Ub is relocated from the intermediate to the substrates [33]. There are about 14 RBR-type E3s encoded in the human genome [40]. These ligases possess Zn2+-binding RING domains (RING1 and RING2). The RING2 domain contains an active site C which alike the HECT-type E3s, is absent in the RING-type E3s. Thus, RBR-type E3s appear to be RING-HECT hybrid in its sequence and domain structure. Catalytically, it adopts similar two-step mechanism as the HECT-type E3s to ligate Ub to substrate proteins [41]. Specifically, the RING1 provides a binding site for the E2-Ub conjugate, and the Ub moiety is firstly transferred to the active C site of RING2 to form a covalent E3-Ub intermediate. During the second step, RING2 mediates ligation of the Ub moiety to the substrate [41]. 

## 3. The Types of Ubiquitination 

The canonical ubiquitination linkage types include monoubiquitination (addition of one Ub monomer at a single K residue), multi-monoubiquitination (simultaneous monoubiquitination at multiple K residues) and polyubiquitination (addition of a Ub chain in which Ub moieties are sequentially linked to a K residue of the existing Ub) [42]. Ub altogether possesses seven K residues (K6, K11, K27, K29, K33, K48 and K63), and any of these Ks can be a site of chain linkage [5,43]. In some cases, the M1 residue of Ub could also be used to form a linear poly-Ub chain. Generally, a poly-Ub chain is homotypic since all Ub moieties in the chain provide the same residue for linkage. Notwithstanding, multiple linkage types can simultaneously arise in a single poly-Ub chain. On the other hand, atypical branched linkages can be formed in which more than one K residues of a single Ub moiety are involved in linkage formation at the same time [44,45]. To date, all the linkage types have been described in eukaryotic cells [7,46]. They are implicated in regulating the fate and activities of the substrate proteins [7,46]. The best-known role of ubiquitination is to serve as a "death label" for substrate protein degradation. Mono-Ub, K6-, K11-, K29- and K48-linked poly-Ub chains are signals which drive proteins for proteasome or lysosome-mediated proteolysis [47,48]. In addition, ubiquitination could also serve as a nonproteolytic signal to regulate the activity and sub-cellular localization of substrate proteins. For instance, receptor interacting protein 1 (RIP1) can be modified by K63-linked poly-Ub chain. This modification is required for the interaction of RIP1 with the Transforming growth factor β-activated kinase 1 (TAK1)/TGF-beta-activated kinase 1 and MAP3K7-binding protein (TAB) complex and inhibitors of I-κB kinase (IKK) to activate signal transduction in the NFκB pathway [49,50,51]. Of note, modification by the same linkage type could produce differing outcomes. For example, it is generally believed that K63 linkage acts as a non-degradative signal to modify protein activity. However, the same modification labels Octamer-binding transcription factor-4 (Oct4) for 26S proteasome-mediated degradation in pluripotent stem cells [52,53,54]. K11-linked poly-Ub chain exhibits similar dual roles. It marks substrates for their degradation during cell-cycle process and stem cell differentiation [55,56,57]. In contrast, K11 linkage increases the stability of β-Catenin, hence contributing to the accumulation of the oncogenic β-Catenin in human colon cancer cells [57,58]. On the other hand, a single protein can be modified by different types of ubiquitination, resulting in diverse outcomes. For instance, DNA polymerase processivity factor PCNA (proliferating cell nuclear antigen) can be either monoubiquitinated by the E2-E3 complex RAD6-RAD18 at the K164 residue or further modified with K63-linked poly-Ub chain by another E2-E3 complex MMS2-UBC13-RAD5 [59,60]. These modifications on PCNA determine which DNA damage tolerance (DDT) pathways will be utilized by cells to bypass DNA lesions during replication. Monoubiquitinated PCNA promotes DNA polymerase ζ-dependent (mutagenic) or DNA polymerase η-dependent (error-free) translesion DNA synthesis (TLS), while polyubiquitinated PCNA can initiate template switching (TS) for an error-free lesion bypass [59,61].

## 4. Ubiquitination and Embryonic Stem Cells 

Stem cells exhibit unique "stemness" state that is defined by the ability to self-renew and differentiate to germ lineages. These specialized cells can be found in both adult and embryonic tissues, performing vital functions in cell regenerations, growth and embryo development. Based on their capacity to differentiate, stem cells can be distinguished into four types, namely the totipotent, pluripotent, multipotent or unipotent stem cells. Totipotent stem cells alone can give rise to an entire organism. Such developmental potential resembles that of the fertilized zygote and the blastomeres up till the eight-cell stage [62]. Pluripotent stem cells are not able to produce an organism by themselves. Nevertheless, they can differentiate into all the cell types in an organism. They are best represented by the embryonic stem (ES) cells, embryonic germ (EG) cells or the embryonic carcinoma (EC) cells. Multipotent stem cells can give rise to certain specialized lineage cells. Most of the adult stem cells are multipotent. They include hematopoietic stem cells, mesenchymal stem cells (MSCs), and other adult progenitor cells. The differentiation capacity of unipotent stem cells is restricted since they can only give rise to one type of cells, such as the myoblast. 

Pluripotent ES cells can be derived from the inner cell mass (ICM) of early blastocysts [63]. The embryonic origin and pluripotent potential provides ES cells as a great model for the study of gene function, early embryogenesis and directed differentiation for future cell replacement therapy in clinic. A panel of proteins, including signaling pathway mediators, transcription factors (TFs) and epigenetic regulators, cooperate tightly to form precise regulatory networks orchestrating the stemness of ES cells. Any dosage or activity alteration in these proteins via ubiquitination could impact on ES cell self-renewal and differentiation capacity. As expected, increasing numbers of ubiquitination-related factors have been identified for their important roles in ES cell regulation. 

### 4.1. Regulation of Stemness-Related TFs by Ubiquitination in ES Cells

TFs are the most abundant group of proteins encoded by the mammalian genome [64]. They are characterized by the capability of directly interacting with DNA elements to regulate transcription. In ES cells, a group of TFs comprise a delicate regulatory circuitry that pivotally monitors the stemness-specific gene expression profile. Among them are the core stemness regulator trinity, Oct4, Sex determining region Y (SRY)-related high-mobility group (HMG) Box 2 (Sox2) and Nanog homeobox (Nanog) [65,66,67]. 

Oct4 belongs to the Pit-Oct-Unc (POU) TF family. It contains two main DNA-binding domains, the POU-specific domain (POUs) and POU homeo-domain (POUh), which are separated by a flexible α-helix linker that enables both POUs and POUh to independently interact with DNA targets. These two domains exhibit high evolutionary conservation in sequence and can also mediate Oct4 interaction with other transcriptional regulators [68]. Two other regions, including the N- and C-terminus, are required for transactivation [69]. ES cells are highly sensitive to the dosage of Oct4. Either two-fold induction or reduction of Oct4 results in ES cell differentiation [70]. Hence, stemness maintenance requires a precise regulation of Oct4 level. HECT-type E3 Wwp2 is the first E3 identified capable of ubiquitinating both mouse and human Oct4, promoting the 26S proteasome-mediated degradation [71,72]. Wwp2 belongs to the WW domain-containing Nedd4 subtype of HECT E3s. Moreover, the C-terminal HECT domain, it contains one N-terminal C2 domain and four tandem WW domains in the middle portion [54,72]. Unexpectedly, Wwp2 and Oct4 exhibit a similar, rather than opposite, expression profile during ES cell differentiation [71,73]. This could be because the repression of *Oct4* transcription surmounts Wwp2 downregulation-resulted Oct4 induction in the differentiation process. Importantly, Wwp2-mediated Oct4 repression could impede stemness re-establishment since both *Wwp2* knockout and mutation in the Oct4 ubiquitination site increase the efficiency of somatic cell reprogramming into induced pluripotent stem cells (iPSCs) [74]. Another interesting observation is that the Wwp2-mediated regulation of Oct4 varies among ES cells originated from different sources. In human ES cells (hESCs), WWP2 downregulates OCT4 in a dosage-dependent manner, whereas in mouse ES cells (mESCs), Wwp2 fails in reducing Oct4 unless differentiation occurs [54,71]. Itch is a second HECT-type E3 mediating Oct4 ubiquitination and degradation by 26S proteasome [75]. Although there is evidence showing that *Itch* depletion impairs self-renewal and reduces the efficiency of iPSC formation, whether Oct4 serves as the dominant downstream effector of Itch is unclear [75]. Of note, both Itch and Wwp2 ubiquitinate Oct4 with K63-linked polymer, which serves as classical examples for K63 linkage-driven substrate degradation [54,75]. RING-type E3 TRIM32 is a third ligase capable of mediating Oct4 ubiquitination [76]. However, it is rather unexpected that it regulates Oct4 independent of its enzymatic RING domain [76]. 

Sox2 belongs to the HMG-domain containing Sox TF family [77]. It is important for embryogenesis and ES cell maintenance. Similar with Oct4, Sox2 has dosage-dependent role in ES cells. Either the reduction or induction of its expression results in the loss of ESC stemness [78,79] [80]. Moreover, Sox2 and Oct4 form a binary complex to activate stemness-related genes, while repressing differentiation-promoted transcripts [65,81]. Their close association extends to ubiquitination-mediated regulation. The E3 Wwp2 for Oct4 could also target Sox2 for ubiquitination; hence, resulting in its degradation in mESCs [82]. However, this modification requires a Set7-catalyzed monomethyl signal at Sox2-K119 [82]. Since the activity of Wwp2 toward Oct4 exhibits inconsistency among different-originated ES cells, how WWP2 regulates SOX2 in hESCs remains unclear. A study by Wang et al. shows that APC/C coordinates with a priming E2 UbcH5/UbcH10 and an elongating E2 ubiquitin-conjugating Enzyme E2S (Ube2s) to modify Sox2 with K11-linked poly-Ub chain at Sox2-K123 residue for 26S proteasome-mediated degradation [57]. Furthermore, Ube2s has been shown to reinforce the stemness state through the fine-tuning of the precise level of Sox2 [57]. Under specific induction condition, Ube2s-APC/C-mediated Sox2 modification can increase the efficiency of mesoendoderm formation, while blocking neuroectodermal lineage commitment [82]. It will be of great interest to decipher how Wwp2 and Ube2s-APC/C exert concerted action to fine-tune Sox2 and Oct4 in the process of ES cell maintenance and cell fate commitment.

Nanog was first identified as “ENK” (early embryo specific NK) based on the homolog of its homeodomain to NK protein family [83]. Besides the DNA-binding homeodomain, Nanog contains two additional transactivation domains at the N- and C-terminus, respectively [10]. The C-terminus can be further divided into three sub-regions, namely CD1, tryptophan repeat (WR) and CD2 [10]. In ES cells, Nanog plays a pivotal role in maintaining stemness-specific genetic and epigenetic landscape [77,84,85,86,87]. *Nanog* knockout leads to loss in pluripotency and self-renewal, while Nanog-elevated mESCs impedes chemical-induced differentiation. In hESCs, NANOG promotes pluripotency and inhibits neuroectoderm differentiation [80,84,87,88]. NANOG exhibits high sensitivity to 26S proteasome inhibitor and can be modified by K48- and K63-linked poly-Ub chain in hESCs. Furthermore, F-box and WD40 domain-containing protein 8 (FBXW8) is identified as an E3 ligase for Nanog ubiquitination and degradation. However, this activity is restricted to phosphorylated Nanog at S52/71/78 [89]. *FBXW8* depletion results in impaired self-renewal [89]. On the other hand, ES cells employ compensatory mechanisms to prevent the excessive degradation of Nanog. For example, H2A.Z directly interacts with Nanog to prevent Nanog ubiquitination in mESCs [90]. The deubiquitinase USP21 is able to remove the degradative Ub signal on human and mouse Nanog, hence preventing its degradation [91,92,93].

### 4.2. Regulation of Signal Transduction Pathways by Ubiquitination in ES Cells

Precise regulation of self-renewal and pluripotency requires concerted action between extra- and intracellular signals. In general, the external molecules bind with cell-surface receptors to evoke signal transduction in the cytoplasm and modulate gene activity in the nucleus. Human and mouse ES cells dissimilarly respond to external signals and adopt distinct pathways to maintain their properties [94,95,96]. For instance, Leukemia inhibitory factor (LIF) is specifically required by in vitro culture of mESCs, while the basic fibroblast growth factor (FGF2) signal is employed for human ES cell growth [97,98,99,100,101,102]. Yet, some signaling pathways, such as the bone morphogenetic protein (BMP) and Wnt pathways are required by both types of ES cells.

BMPs belong to the transformation growth factor beta (TGFβ) family, which is widely involved in cell proliferation, differentiation and apoptosis [103]. There are two main types of BMP receptors for over 20 BMPs: type I (including Alk2, Alk3, and Alk6) and type II (BmprII) [85]. Interaction between different receptors determines the specificity and consequences of BMP functions [85]. In general, BMPs bind to receptors, resulting in phosphorylation of downstream effectors, Smad1, Smad5 or Smad8 (receptor-regulated Smad, R-Smad). Two of these phosphorylated R-Smads form a heterotrimer with a common Smad protein, Smad4 (co-Smad), to translocate into the nucleus for transcription regulation [104,105,106,107]. The BMP signals can be inhibited by two inhibitory Smads (I-Smads), Smad6 and Smad7 [108,109]. In mESCs, the BMP/Smad signaling coordinates with the LIF stimulus to sustain stemness. Loss of BMP signal leads to impaired pluripotency and neuroectodermal differentiation [110]. In hESCs, the BMP signal cooperates with OCT4 and the FGF2 signal, respectively, to govern cell fate commitment [80]. Several studies have investigated how ubiquitination regulates the BMP signaling pathway in embryos and multiple types of adult stem cells [111]. Two HECT-type E3 ligases, Smad ubiquitination regulatory factor 11 (Smurf1) and Smurf2 are involved in ubiquitinating Smads. Smurf1 can modify Smad1 and Smad5 for degradation via poly-Ub chain, while Smurf2 preferentially ubiquitinates Smad1 [112,113,114]. The C-terminus of Hsc70-interacting protein (CHIP) is a third E3 ligase regulating Smad degradation. CHIP belongs to U box-type E3s. It can mediate poly-Ub chain formation specifically on Smad1 and Smad5 for 26S proteasome-mediated degradation [50,115]. Moreover, another two HECT-type E3s, WWP1 and NEDD4–2, are also identified for their capability of ubiquitinating and degrading Smad proteins [116]. However, how these E3s regulate ES cell maintenance is unknown. A study by Zhang et al., revealed the mechanism underlying Smad7 ubiquitination in mESCs. It reported that RING-type E3 RNF12 promotes Smad7 degradation via polyubiquitination. Inhibition of RNF12 results in Smad7 accumulation, which rescues BMP-triggered neuroectodermal differentiation [117].

**Figure 2:**
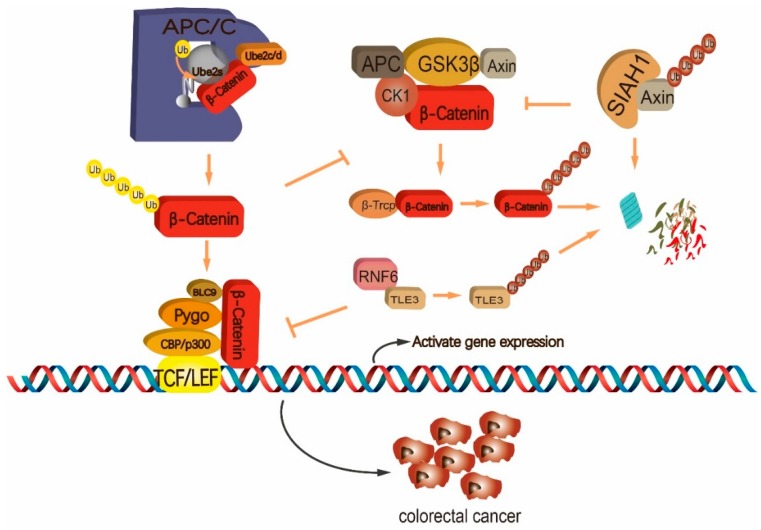
Ubiquitination-monitored regulation of the Wnt/β-Catenin pathway in colorectal cancer (CRC) cells. UBE2S coordinates with the E3 complex anaphase-promoting complex/cyclosome (APC/C) to mediate K11-linked ubiquitin polymer on β-Catenin, which prevents beta-transducin repeat containing E3 ubiquitin protein ligase (β-TrCP)-orchestrated degradation of β-Catenin. RING-type E3 ring finger protein 6 (RNF6) ubiquitinates the inhibitor of β-Catenin, transducin-like enhancer of split 3 (TLE3), for degradation, which enhances the transcriptional activity of β-Catenin. SIAH1 ubiquitinates Axin for degradation, and thus blocks β-TrCP-mediated recognition and degradation of β-Catenin. Arrows represent enhancement and T-bars represent inhibition.

The Wnt signaling cascade is activated by the binding of Wnt ligands to Frizzled receptor and its co-receptor, low-density-lipoprotein-related protein 5/6 (LRP5/6). Upon ligand binding, β-Catenin is dissociated from the Axin destructive complex and enters the nucleus to interact with T-cell factor/lymphoid enhancer factors (Tcf/Lef) for transcription regulation [118,119,120]. Under the normal serum culture condition, activation of the Wnt pathway by CHIR99021, an inhibitor against glycogen synthase kinase 3 (GSK3), confers mESCs resistance to LIF withdrawal-induced differentiation [121]. Consistently, increased β-catenin accumulation leads to impaired ES cell differentiation [122]. In addition, in a serum-free culture medium, CHIR99021 combines with the MEK1/2 inhibitor PD0325901 and/or LIF to endow mESCs with a naive pluripotency [123]. In hESCs, either excessive induction or repression of the Wnt/β-catenin signaling results in loss of stemness [124]. As the key effector of the Wnt signal, β-catenin is precisely regulated to guarantee appropriate activation status of the pathway. Without Wnt stimulus, β-Catenin is associated with the Axin destructive complex which is composed of multiple factors, mainly including Axin, adenomatous polyposis coli (APC), GSK3 and casein kinase 1 (CK1). β-Catenin is modified by CK1- and GSK3-mediated sequential phosphorylation at its S45, S33, S37 and T41 residues [125,126,127]. Consequently, the phosphorylated β-Catenin is recognized by the E3 complex Skp1/Cul1/F-box^β-TrCP^ for K48-linked polyubiqutination and proteasomal degradation [118,128,129,130,131]. Upon Wnt ligand binding, LRP6 is phosphorylated, which leads to GSK3 inhibition, collapse of the Axin complex and β-Catenin dissociation. Axin is further captured by RING-type E3 SIAH1/2 for ubiquitination and degradation (Figure 2) [132]. Dissociated β-Catenin is modified by Ube2s-APC/C-mediated polyubiquitination with a K11 linkage (Figure 2) [58]. This modification allows β-Catenin to avoid the β-TrCP-mediated degradative ubiquitination, hence enhancing its stability [58]. Importantly, the activity of Ube2s-APC/C toward β-Catenin promotes mESC commitment to the mesoendoderm lineage [58].

### 4.3. Regulation of ES Cell-Related Epigenetic Regulators by Ubiquitination

ES cells exhibit unique chromatin architecture, which contributes to the stemness maintenance and at the same time, allows ES cells to rapidly respond to differentiation signals [133,134]. The ES cell-specific chromatin landscape is largely monitored by histone modifications, such as methylation, acetylation and ubiquitination [135]. For example, the nucleosomes of ES cell chromatin contain a characteristic bivalent domain simultaneously possessing transcriptional active H3K4 and the repressive H3K27 methylation signals [136]. The K120 residue of Histone H2B can be monoubiquitylated (H2B-K120Ub1) by the RING-type E3 RNF20/RNF40 in mammalian cells, which is generally related with gene activation [137,138,139]. Depletion of *RNF20* can confer mESC resistance to induced differentiation [140]. H2B-K119 is another monoubiquitination site which is closely related to the ES cell identity. H2B-K119Ub1 is dominantly achieved by the Ring1A and Ring1B subunits of Polycomb repressive complex 1 (PRC1). Knockdown of *Ring1A/1B* leads to reduction in H2B-K119Ub1 and ES cell differentiation [141,142,143,144,145]. RING-type E3 Dzip3 can also mediate H2B-K119Ub1 formation. However, its activity appears to be restricted to the promoter regions of several differentiation-related genes [146]. Interestingly, a cross-talk exists between the H2B-K119Ub1 signal and the bivalent domain to co-operatively monitor gene activity. They co-occupy differentiation-related genes for its enhanced repression, resulting in the reinforcement of the stemness state [146]. Reduction of the H2B-K119Ub1 signal upon *Ring1A/1B* depletion results in inefficient inhibition of bivalent genes and the lost of pluripotency [147].

## 5. Ubiquitination-Mediated Regulation of Cancer Development

Malignant cancers display uncontrolled growth, capability of invasion and mobility, intratumoral heterogeneity, and high recurrence rate [148]. Mounting evidences show the critical roles of ubiquitination-related factors in regulating tumorigenesis and malignancy. This part of the review focuses on two human cancers, the colorectal cancer (CRC) and osteosarcoma (OS), highlighting recent the recent progresses in the field.

Malignant CRC displays a high rate of incidence and mortality [147]. The lesion sites can be found in colon, rectum and appendix. The molecular signatures governing CRC occurrence and progression can mainly be grouped into three categories: (1) Genomic instability induced by inactivation of tumor suppressors (*APC*, *TP53*, *SMAD4*) or activation of proto-oncogene *KRAS*; (2) Microsatellite instability (MSI) caused by abnormal DNA mismatch repair (MMR); (3) Abnormal transcription due to hypermethylation in CpG islands [149,150,151,152]. Moreover, other mutations can also be observed in CRC. For example, about 10% of CRC patients bear mutation in tumor suppressor von Hippel-Lindau (VHL) [153]. VHL forms a CRL2^VHL^ E3 complex with elongin B/C, RBX1, ROC1 and CUL2 to ubiquitinate Hypoxia Inducible Factor 1α (HIF-1α) for proteolysis in an oxygen-dependent manner [154,155,156]. HIF-1α promotes angiogenesis, cell metabolism and survival under hypoxia condition and VHL mutation exhibits a close correlation with abnormal accumulation of HIF-1α in CRC cells [153,157,158]. Of note, among all these mutations, *APC* mutation affected about 90% of CRC patients [151,159]. Since APC is required by β-TrCP-mediated degradative ubiquitination of β-Catenin in the Wnt pathway, abnormal β-Catenin accumulation is always detected in the CRC patients. Moreover, about 10% of CRC patients contain genetic mutation in *β-Catenin* whose product maintains the ability in mediating Wnt signal transduction but fails to be modified and degraded by the Axin-β-TrCP cascade [160]. Therefore, excessive β-Catenin accumulation is considered as a fundamental event in CRC. However, genetic mutation-caused inefficiency in β-Catenin degradation could be conquered by RING-type E3 TNF receptor-associated factor 6 (TRAF6). It ubiquitinates autophagy sensor LC3B via K63 linkage, which allows LC3B to recognize β-Catenin and drive it for autophagic degradation, independent on the activity of APC and β-TrCP ligase (Figure 2) [161]. Besides genetic mutations, some ubiquitination factors contribute to the excessive activation of the Wnt/β-Catenin pathway in CRC cells (Figure 2). E2 UBE2S coordinates with the E3 complex APC/C to mediate K11-linked ubiquitin polymer on intact β-Catenin, which prevents β-TrCP-orchestrated degradation and enhances β-Catenin accumulation in CRC cells (Figure 2) [58,162]. Moreover, RING-type E3 ring finger protein 6 (RNF6) indirectly enhances the activity of β-Catenin through suppressing its inhibitor, transducin-like enhancer of split 3 (TLE3) [162]. TLE3 inhibits the transcriptional activity of β-Catenin [163]. RNF6 modifies TLE3 via ubiquitination for proteolysis, which promotes CRC malignancy and recurrence (Figure 2) [162]. On the other hand, ubiquitination-mediated regulation of CRC progression is not restricted to the Wnt signaling, but widely involved in various important cellular processes, such as stem cell differentiation, autophagy, epithelial–mesenchymal transition (EMT) and epigenetic regulation of transcription [164,165,166]. The E3 ubiquitin ligase FBXW7 is involved in regulating stem cell proliferation and commitment in normal intestine and colon tissues [167,168]. *FBXW7* deletion in mouse intestine impairs stem cell differentiation and promotes tumorigenesis [167]. In human CRC cells, FBXW7 represses EMT-mediated metastasis through ubiquitinating transcription factor ZEB2 for degradation [169]. However, disabled *FBXW7* mutation is detected in CRC patients and FBXW7 exhibits a decreased expression in lesion sites, which deprives of FBXW7-mediated CRC repression [170,171,172]. In addition, *FBXW7* mutation results in accumulation of myeloid cell leukemia 1 (MCL1) that is another substrate of FBXW7-mediated ubiquitination [173,174]. MCL1 belongs to pro-survival BCL2 family and is involved in mitochondrial apoptosis [175]. *FBXW7* mutation-induced MCL1 accumulation results in chemotherapy insensitivity of CRC cells in clinic [176]. HECT-type E3 HECTH9 exhibits a very low expression in normal gut epithelium and induced expression in CRC cells [177]. It modifies stem cells-related regulator C-MYC with K63-linked poly-Ub chain to monitor C-MYC transcriptional activity and thus promotes CRC cell proliferation [177]. Contradictorily, Hecth9 does not promote tumor growth but acts as a repressor of CRC development in mice [178,179]. On the other hand, several studies provide clues for uiquitination-monitored epigenetic regulation of CRC progression. First of all, most CRC patients possess decreased RNF20/RNF40 expression and global loss in H2BK120ub1 signal accompanying poor therapeutic outcome [180,181]. Defect in H2BK120ub1 results in series of disorders, such as proto-oncogene activation, replication stress and impaired DNA damage repair genome instability and subsequent tumorigenesis [182,183,184,185]. Therefore, abnormity in H2BK120ub1 signal could be employed to develop novel strategy for CRC treatment in future. Chromatin-remodeling factor special AT-rich sequence-binding protein-1 (SATB1) promotes colon tumorigenesis [186,187]. SMURF2 ligase mediates SATB1 ubiquitination and promotes its degradation [188]. SMURF2-mediated SATB1 modification effectively inhibits CRC progression and confers sensitivity of CRC cells to conventional chemotherapy agents [188]. Recently, SMURF2 is suggested as a putative prognostic marker for MSI-free CRC patients [189]. Of note, besides the factors which were discussed above, additional ubiquitination-related factors have been identified to play critical roles in the regulation of CRC. They are summarized in Table 1.

OS is a malignant bone tumor with high incidence in children and adolescents [227]. In spite of relatively low prevalence, OS results in high death rate due to poor therapeutic effect in clinic [228]. OS displays intratumoral heterogeneity and stem cell properties possibly due to defect in osteoblast differentiation from MSCs [229,230,231]. In the normal commitment process from MSC to terminal osteocytes, different types of mid-term cells are transiently produced at corresponding differentiation stages, including committed osteoprogenitor, proosteoblast, early osteoblast, mature osteoblast and osteocyte. These cells can be characterized by marker gene expression. For example, in undifferentiated MSCs, the BMP/SMAD signal is highly activated that dominantly induces the expression of inhibitors of differentiation (IDs) to support cell proliferation [232,233,234]. Upon differentiation, the BMP/SMAD pathway is inactivated while the markers of pro-osteoblasts, *RUNX2* and *OSTERIX*, are induced [232,233,234,235]. Mature osteoblasts and osteocytes can be featured by the expression of Osteocalcin (OC) and Osteopontin (OPN, SPP1) [232]. Although there are evidences showing OSs can be originated from terminally differentiated osteoblast, immature cells can always be detected in OS expressing semidifferentiated cell markers and even ES cell markers, such as Oct4 and Sox2 [236,237,238]. To suppress the poor differentiation properties through eliminating the excessive expression of stem cell markers could serve as an avenue for a better treatment of OS. This is well supported by the study of Zhang et al. [238]. They exploit SMURF1 to inhibit the BMP/SMAD signal, which successfully drives OS cells to re-enter the process of differentiation. More importantly, the differentiated OS cells are conferred sensitivity to chemotherapeutic agents [238]. In details, SMURF1 cooperates with E2 complex UBCH5B-UEV1A to modify SMAD1 with poly-Ub chain, which destines SMAD1 for 26S proteasome-mediated degradation [238]. Interestingly, SMURF1 can also serve as the E3 of RUNX2. It mediates RUNX2 ubiquitination for degradation, which is important for osteoblast differentiation [113]. Therefore, SMURF1 possibly possess double effect on OS repression especially for the subtypes highly expressing both SMAD1 and RUNX2. Interestingly, HECT-type E3 WWP1 is also involved in regulating RUNX2. It is recruited by zinc finger-containing adaptor Schnurri-3 (SHN3) to interact with RUNX2 and mediate its polyubiquitination for degradation [239]. This activity is involved in regulating extracellular matrix mineralization in the process of adult bone formation [239]. However, their impact on OS growth and metastasis is unclear. E3 MDM2 is involved in maintaining the stem cell properties of OS cells. It ubiquitinates retinoic acid receptor alpha (RARα) for proteasomal degradation, which impedes retinoic acid (RA)-induced OS differentiation and promotes malignancy [240] Therefore, inhibition of MDM2 could serve as a possible avenue to effectively suppress OS progression. On the other hand, increasing numbers of ubiquitination factors have been identified for their roles in regulating OSs independent of cell differentiation. For instance, CUL4B displays an elevated expression in OS cells to promote proliferation and inhibit apoptosis [241]. It cooperates with three additional proteins, RING-box protein 1 (RBX1), DNA damage binding protein 1 (DDB1), and DDB1- and CUL4-associated factor 13 (DCAF13), to form the CRL4B^DCAF13^ E3 complex. Via the DCAF13 subunit, this complex can specifically recognize the tumor repressor, phosphatase and tensin homolog deleted on chromosome 10 (PTEN), and exert CUL4/RBX1-mediated ubiquitination of PTEN for degradation [242]. Another study shows that CUL4B/RBX1/ DDB1 can coordinate with DCAF11 to form the CRL4B^DCAF11^ E3 complex that ubiquitinates the cyclin-dependent kinase (CDK) inhibitor p21^Cip1^ and promotes its degradation. This activity is required by the proliferation of OS cells [243]. The RING-type E3 c-Cbl serves as an OS repressor [244]. It is downregulated in OS cells, and targets receptor tyrosine kinase (RTK) for degradation via ubiquitination. Overexpression of c-Cbl results in excessive degradation of RTK, which inhibits the proliferation and metastasis of OS cells [244]. Tryptophane-aspartic acid (WD) repeat and SOCS box containing 1 (WSB1) ligase, elongin B, C-Cullin5 and Rbx1 form a multiple-unite E3 complex, involved in regulating multiple types of cancers [245,246,247]. In OS cells, this E3 complex promotes hypoxia-enhanced metastasis through modifying Rho guanosine diphosphate dissociation inhibitor 2 (RhoGDI2) with poly-Ub chain for proteasome-mediated degradation [248]. Moreover, other ubiquitination factors have also been reported for their involvement in regulating OS biology, such as UBE2T, Adaptor Speckle-type pox virus and zinc finger protein (POZ) protein (SPOP) and HECT domain and ankyrin-repeat-containing E3 ubiquitin-protein ligase 1 (HACE1) [249,250,251]. These factors could form a network to globally regulate the onset and progression of OS cells.

## 6. Concluding Remarks and Perspectives

Ubiquitination monitors the longevity and activity of proteins. A myriad of protein-dependent cellular processes, including cell cycle progression, gene transcription, chromatin remodeling, signaling transduction and endocytosis, are precisely dominated by the ubiquitination machinery. Mounting evidences show the close correlation between ubiquitination dysregulation and human diseases. Recent studies start a promising paradigm to utilize the "death label" produced by the ubiquitination system to remove disease-causing proteins. Ubiquitination mediators, especially E3s, act as putative targets to develop novel therapeutic approaches with higher effectiveness and fewer side effects [252]. Excitingly, the inhibitors against several E3s, such as APC/C, MDM2 and SKP2, are being evaluated for cancer treatment in the preclinical or clinical stages [253,254,255,256,257,258]. Moreover, proteasome inhibitor drugs, Bortezomib and Carfilzomib, have been successfully applied in the clinic for the treatment of human cancers [259]. However, to extend this paradigm for more types of cancers requires a comprehensive understanding of the ubiquitination-mediated regulatory mechanisms. We need to identify dominant ubiquitination factors related to tumorigenesis and malignancy. Moreover, it is also challenging to develop effective therapeutic molecules specifically targeting these factors. In pluripotent ES cells, previous studies largely focused on the transcriptional regulatory network, while the information about how ubiquitination regulates the self-renewal and pluripotency is much more limited. Specific questions of interest include how ubiquitination coordinates with other types of post-translational modifications, such as methylation, acetylation and phosphorylation, to globally monitor the properties of ES cells and cell fate specification. To dissect these puzzles could open a new arena for ES cell application and disease therapy in future.

## Figures and Tables

**Table 1:** Ubiquitination factors involved in regulating CRC.

E2	E3	Function	Reference
UBC3 /UBC4	β-TrCP	1. β-TrCP ubiquitinates phosphorylated IκB for degradation, which enhances the NF-κB signaling. Increased β-TrCP is associated with an enhanced NF-κB signaling in CRC. 2. β-TrCP ubiquitinates β-Catenin via K48-linked poly-Ub chain for proteasomal degradation, which suppresses CRC progression.	[190,191,192]
UBCH5 /UBCH10/UBE2S	APC/C	1. APC/C^cdc20^ ubiquitinates Conductin for degradation during mitotic exit, which regulates the Wnt/β-catenin signaling and CRC cell growth. 2. UBE2S collaborates with the APC/C complex to stablize β-Catenin via K11-linked polyubiqutination. This activity enhances CRC proliferation and metastasis.	[58,193]
UBCH5	HECTH9	HECTH9 modifies C-MYC with K63-linked poly-Ub chain to promote CRC cell proliferation. The expression of HECTH9 is increased in the cancer tissues of CRC patients.	[177]
UBCH5	S-phase kinase protein 2 (SKP2)	SKP2 ubiquitinates p27Kip1 for degradation in CRC cells. Elevated expression of SKP2 and reduced expression of p27Kip1 is associated with poor prognosis and decreased survival of CRC patients.	[194,195,196,197]
UBCH5B/ UBE2S	von Hippel-Lindau protein (pVHL)	VCB-Cul2 ubiquitinates HIF-1α for degradation under hypoxic conditions, which suppresses CRC malignancy.	[156,198,199,200]
UBCH5B	X-chromosome-linked IAP (XIAP),	1. XIAP ubiquitinates active caspase-3 for degradation to suppress apoptosis. Inhibition of XIAP increases the sensitivity of *PIK3CA*-mutated CRC cells for induced cell death. 2. XIAP monoubiquitinates TLE, which promotes β-catenin-TCF association and enhances activation of the Wnt pathway in CRC cells.	[201,202,203]
UBCH6	RNF20/RNF40	RNF20/RNF40 monoubiquitinates H2B-K120, which is required by transcription regulation. Loss of H2BK120ub1 is associated with poor therapeutic outcome in CRC.	[181,182,204]
UBC9	E6-AP	E6-AP coordinates with UBC9 to ubiquitinate SOX9 for degradation, which may repress Sox9-enhanced CRC malignancy.	[205]
UBC13/ UEV1A	Tumor necrosis factor receptor-associated factor 6 (TARF6)	1. TARF6 ubiquitinates IKK via K63-linked poly-Ub chain to promote the NF-κB signaling pathway. High expression of TRAF6 is associated with a decreased survival of CRC patients. 2. TARF6 stabilizes hypoxia-inducible factor (HIF)–1a through K63-linked polyubiquitination, which promotes angiogenesis and growth of CRC. 3. TRAF6 ubiquitinates LC3B via K63 linkage, which allows LC3B to recognize β-Catenin and drive it for autophagic degradation. This activity is involved in inhibiting the metastasis of CRC cells.	[161,206,207,208,209]
	c-IAP	c-IAP is upregulated in CRC patients with a reduced survival.	[210]
	FBXW7	FBXW7 ubiquitinates ZEB2 and MCL1 for degradation, which is involved in regulating the malignancy and therapy resistance of CRC cells. Somatic mutations in *FBXW7* is detected in CRC patients.	[169,171,172,176,211,212]
	Human upstream regulatory element binding protein 1 (hUREB1)	hUREB1 down-regulates p53 through ubiquitination in CRC cells. Increased expression of hUREB1 is correlated with p53 destabilization in CRC patients.	[213]
	MDM2	MDM2 ubiquitinates p53 for degradation. Increased expression of MDM2 is correlated with negative expression of p53 in CRC patients.	[214,215]
	RNF4	RNF4 ubiquitinates and stabilizes multiple oncoproteins, such as c-Myc and β-Catenin. Elevated expression of RNF4 is correlated with CRC tumorigenesis.	[216,217,218]
	RNF6	RNF6 enhances the interaction between β-Catenin and TCF4/LEF through ubiquitinating transducin-like enhancer of split 3 (TLE3) for degradation, which promotes CRC cell growth and metastasis.	[162]
	RNF14	RNF14 activates the Wnt pathway through interacting with TCFs to promote β-Catenin recruitment, which promotes CRC cell growth.	[219,220]
	Ubiquitin E3 ligase ring finger 43 (RNF43)	RNF43 ubiquitinates Frizzled (FZD) and LRP6 for degradation. About 18% of CRC patients bear *RNF43* truncating mutation.	[221,222,223]
	Tripartite motif (TRIM3)	TRIM3 enhances the stability of p53 and suppresses CRC development.	[224]
	TRIM15	TRIM15 serves as a putative CRC suppressor, which inhibits CRC cell growth and metastasis.	[225]
	TRIM29	TRIM29 is upregulated in aberrant crypt foci in human colon and serves as a putative biomarker for CRC diagnosis.	[226]

## Abbreviations

APC	Adenomatous polyposis coli
APC/C	Anaphase-promoting complex/cyclosome
BMP	Bone morphogenetic protein
CDK	Cyclin-dependent kinase
CHIP	Hsc70-interacting protein
CK1	Casein kinase 1
CRC	Colorectal cancer
CRLs	Cullin-RING ligases
DCAF13	DDB1- and CUL4-associated factor 13
DDB1	DNA damage binding protein 1
DTT	DNA damage tolerance
E3	Ub ligase
EC cells	embryonic carcinoma cells
EG cells	Embryonic germ cells
EMT	Epithelial–mesenchymal transition
ENK	Arly embryo specific NK
ES cells	Embryonic stem cells
FBXW8	F-box and WD40 domain-containing protein 8
FGF2	Fibroblast growth factor
FZD	ubiquitinates Frizzled
GSK3	Glycogen synthase kinase 3
H2B-K120Ub1	K120 residue of Histone H2B can be monoubiquitylated
HACE1	HECT domain and ankyrin-repeat-containing E3 ubiquitin-protein ligase 1
HECT	homologous to E6-AP COOH terminus
hESCs	Human ES cells
HIF-1α	Hypoxia inducible factor 1α
hUREB	Human upstream regulatory element binding protein
ICM	Inner cell mass
IDs	Inhibitors of differentiation
IKK	Inhibitors of I-κB kinase
iPSCs	Induced pluripotent stem cells
LIF	Leukemia inhibitory factor
LRP5/6	low-density-lipoprotein-related protein 5/6
MCL1	Myeloid cell leukemia 1
MDM2	Mouse double minute 2
mESCs	Mouse ES cells
MMR	Mismatch repair
MSCs	Mesenchymal stem cells
MSI	Microsatellite instability
NADH	Nicotinamide adenine dinucleotide
OC	Osteocalcin
Oct4	Octamer-binding transcription factor-4
OPN/SPP1	Osteopontin
OS	Osteosarcoma
PCNA	Proliferating cell nuclear antigen
POUh	POU homeo-domain
POUs	POU-specific domain
PRC1	Polycomb repressive complex 1
PTEN	Phosphatase and tensin homolog deleted on chromosome 10
pVHL	von Hippel-Lindau protein
RA	Retinoic acid
RARα	Retinoic acid receptor alpha
RBR	RING between RING
RBX1	RING-box protein 1
RhoGDI2	Rho guanosine diphosphate dissociation inhibitor 2
RING	Really Interesting New Gene
RIP1	Receptor interacting protein 1
RTK	Receptor tyrosine kinase
RNF6	RING-type E3 ring finger protein 6
RNF43	Ubiquitin E3 ligase ring finger 43
SATB1	Special AT-rich sequence-binding protein-1
SCF	Skp1/Cul1/F-box
SHN3	Zinc finger-containing adaptor Schnurri-3
Smurf1	Smad ubiquitination regulatory factor 11
Sox2	SRY-related HMG Box 2
SPOP	Speckle-type POZ protein
Tcf/Lef	T-cell factor/lymphoid enhancer factors
TLS	Translesion DNA synthesis
TRAF6	NF receptor-associated factor 6
Ub	Ubiquitin
Uba/E1	Ub-activating enzyme
UBC/E2	Ub-conjugating enzyme
Ube2s	E2 ubiquitin-conjugating Enzyme E2S
WSB1	WD repeat and SOCS box containing 1
